# Giant adrenal pseudocyst removed using robot‐assisted surgery

**DOI:** 10.1002/iju5.12571

**Published:** 2023-01-12

**Authors:** Takayuki Ohzeki, Hiroyuki Kita, Remon Kunishige, Taiji Hayashi, Tsukasa Nishioka

**Affiliations:** ^1^ Department of Urology Izumi City General Hospital Izumi Osaka Japan

**Keywords:** adrenal, giant pseudocyst, laparoscopic adrenalectomy, open adrenalectomy, robot‐assisted surgery

## Abstract

**Introduction:**

Adrenal cysts are relatively rare and often asymptomatic. Surgical treatment is indicated for symptomatic cases with cysts >6 cm, suspected bleeding, and those that cannot be distinguished from malignant illness based on imaging findings. There have often been cases of giant cysts that were difficult to treat using laparoscopic surgery.

**Case presentation:**

A 39‐year‐old woman presented with fever and upper abdominal pain. Abdominal computed tomography and magnetic resonance imaging revealed a 95 × 80‐mm left adrenal cyst. As malignant disease could not be ruled out, and the patient was symptomatic, we opted for robot‐assisted left adrenalectomy. The pathological findings indicated an adrenal pseudocyst.

**Conclusions:**

This is the second report of the successful robot‐assisted removal of a giant adrenal cyst.

**Abbreviations & Acronyms**
CRP: C‐reactive protein
CT: computed tomography
HE: hematoxylin eosin
MRI: magnetic resonance imaging

**Keynote message**

No consensus exists on the surgical approach to giant adrenal tumors. Robot‐assisted adrenalectomy has significant advantages for adrenal cysts and tumors >6 cm where malignancy cannot be ruled out.

## Introduction

Adrenal cysts are relatively rare. A large population‐based study on adrenal tumors in 2017 reported a sex‐ and age‐standardized prevalence rate of patients with adrenal tumors of 532/100 000 inhabitants, with most adrenal tumors being incidental adrenocortical adenomas <40 mm in patients aged >40 years.^1^ Most adrenal cysts are asymptomatic, and large cysts have few subjective symptoms. Compression of the cyst causes upper gastrointestinal symptoms, constipation, and abdominal mass. Surgical treatment is indicated when the patient has a cyst ≥6 cm^2^, ^3^; however, no consensus exists on the surgical approach. There have been few reports on robotic surgery for giant adrenal tumors. Herein, we report our experience with the robot‐assisted removal of a giant adrenal cyst.

## Case presentation

A 39‐year‐old woman sought medical attention in April 2022 for the chief complaint of dull upper abdominal pain and fever up to 38°C lasting approximately 5 days. She had a medical history of Graves' disease without other complications and a family history. Abdominal ultrasound findings suggested a left upper abdominal cyst, and she was admitted for close examination and treatment.

Patient condition at admission was as follows: Height, 165 cm; weight, 50 kg; body mass index, 18.4; blood pressure, 145/54 mmHg; heart rate, 87 bpm; body temperature, 38.8°C. The abdomen was flat and soft, with tenderness in the upper abdomen. However, the mass was not palpable. Endocrinological and hematological examinations revealed no abnormalities except a mildly elevated CRP level (5.9 mg/dL).

**Fig. 1:**
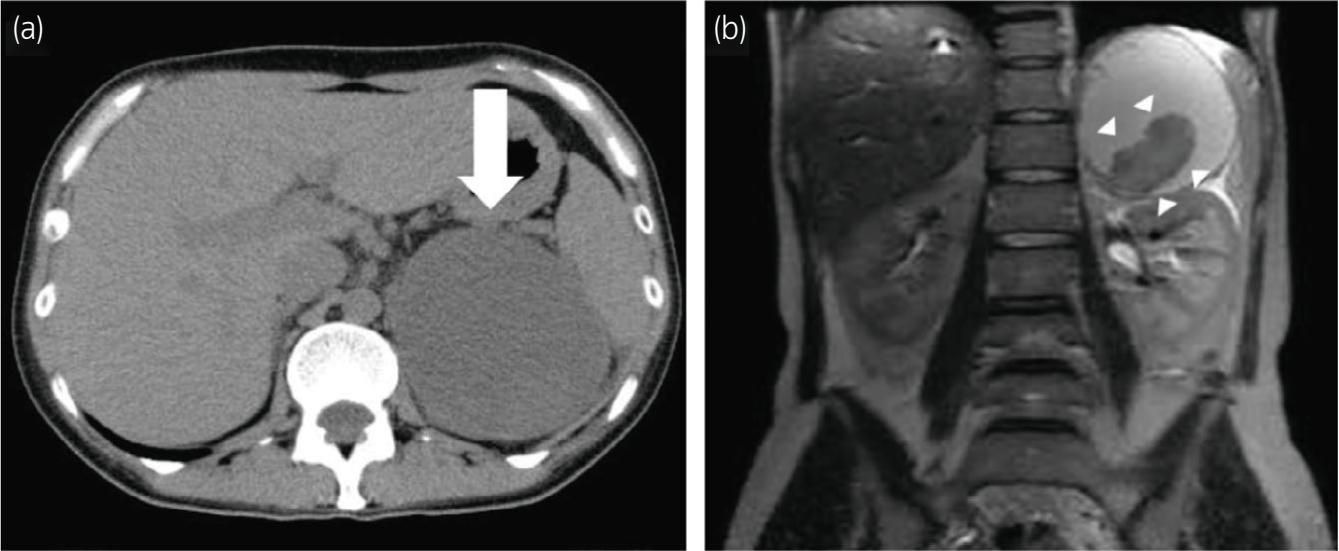
(a) CT image showing a 95 × 80 mm sized cyst on the rostral side of the kidney, and the inside of the cyst was almost homogeneous and water dense (➡). (b) T2‐weighted magnetic resonance image showing high signal with a 52‐mm internal tumor and septal wall (▵).

Plain abdominal CT revealed a 95 × 80‐mm cyst in the left adrenal gland; the left kidney was largely displaced to the caudal side. The cyst interior was uniform with high water density (Fig. 1a). The cyst contents indicated hyperintensity on both T1‐ and T2‐weighted abdominal MRI. Highly viscous liquid or bleeding was suspected. A septum and a 52‐mm solid component were found inside the cyst (Fig. 1b).

**Fig. 2:**
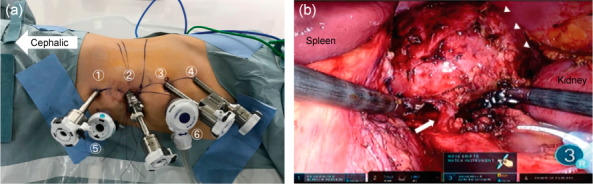
(a) Ports for robotic adrenalectomy. ① 8 mm, left hand ② 12 mm, camera ③ 8 mm, right hand ④ 8 mm, 3rd left hand ⑤ 12 mm, assistant ⑥ 12 mm, assistant (air seal™). (b) A blood vessel (➡) flowing into a giant cyst (▵) was observed, which was thought to be the central vein of the adrenal gland. Normal adrenal tissue was confirmed adjacent to the cyst wall.

The patient was diagnosed with an adrenal cyst, and abdominal MRI findings could not rule out primary‐onset malignancy. Therefore, we decided to resect the mass surgically. The DaVinci Xi system (Intuitive Surgical; 4 ports, and 2 assistant ports, Fig. 2a) was used. Blood loss was minimal, operating time 2 h 33 min, and console time 1 h 37 min. A transperitoneal approach identified a retroperitoneal cyst in the upper pole of the kidney. Although mild adhesion was observed between the mesentery and tumor tissue (Fig. 2b), we managed to remove only the cystic lesions. The left adrenal gland had become a single mass with the tumor and was thus co‐resected.

**Fig. 3:**
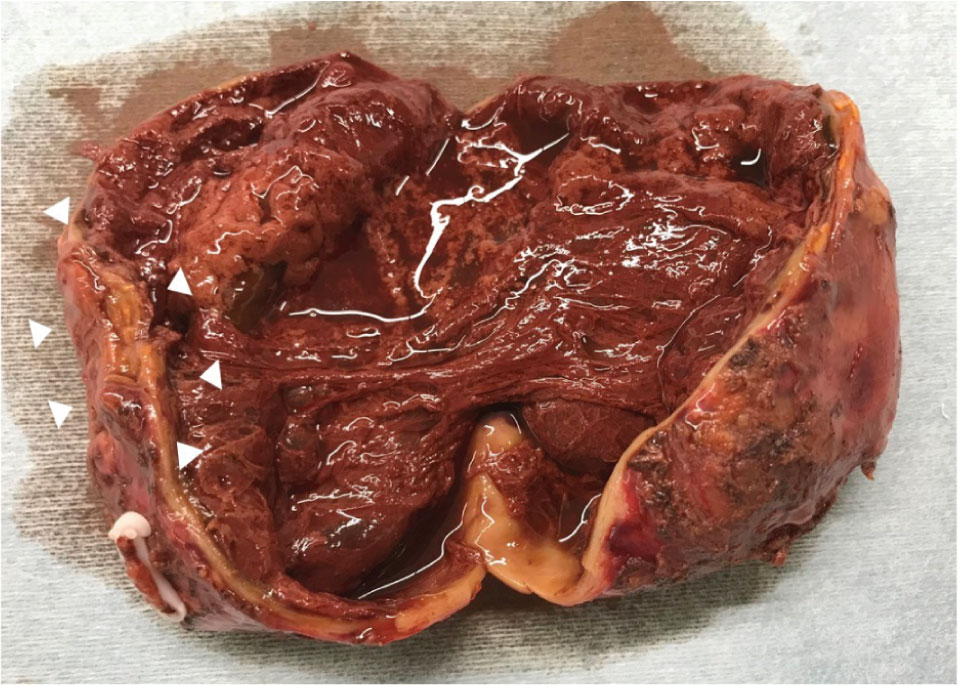
Adhesion of normal adrenal tissue (▵) next to the cyst wall. Unilocular cyst formation was observed on the cut surface of the tumor, and an accumulation of a dark red‐like substance was noted on the inner surface. There was no solid tumor.

**Fig. 4:**
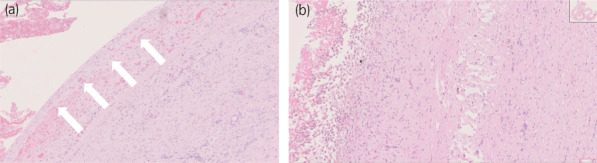
(a) A cyst with fibrous connective tissue wall with vitrification and no epithelial content lining the lumen (➡) (hematoxylin eosin [HE] stain, ′66). (b) In addition to bleeding and adhesion of clotting components in the lumen, infiltration of inflammatory cells and aggregation of pigment‐phagocytic macrophages were observed (HE stain, ′84).

A gross pathological examination of the resected specimen revealed unilocular cyst formation with no solid tumors. Accumulation of a dark red‐like substance was noted on the inner surface (Fig. 3). Pathological examination showed normal adrenal tissue adhered to the cyst wall. Besides bleeding and adhesion of clotting components in the lumen, infiltration of inflammatory cells and aggregation of pigment‐phagocytic macrophages were observed. The patient was diagnosed with adrenal pseudocyst as this was a hemorrhagic cyst without epithelial coverage (Fig. 4). Postoperative fever resolved, CRP was negative, and the patient was discharged on postoperative day 9, with good postoperative progress.

## Discussion

Adrenal cysts, including pseudocysts, are rare tumors, often found as incidentalomas.^4^ Most adrenal cysts are asymptomatic, and the large cysts have few subjective symptoms due to endocrinological inactivity. Acute abdomen may occur due to bleeding in the cyst, which alerted our patient to seek medical attention.

In this case, blood pressure was normal, with no evidence of an endocrinologically suspected functional tumor. We preoperatively diagnosed the patient with an adrenal cyst based on MRI and multi‐slice CT results. Differential diagnoses included retroperitoneal liposarcoma, cystic pheochromocytoma, and adrenal metastases. Around 7% of adrenal cysts are suspected to be malignant; therefore, differentiation from benign cysts is important.^2^ When the inside of the cyst is heterogeneous and CT values are high, calcification is present, or the cyst wall thickness is ≥5 mm, active close inspection is needed to determine if the disease is malignant.^3^ If the patient is asymptomatic with a non‐functional adrenal cyst, simply monitoring the patient is sufficient. However, surgical treatment is indicated when the patient is symptomatic, has a cyst ≥6 cm, or when it is difficult to determine whether it is a malignant disease based on image findings.^2^, ^3^

Surgical approaches are mainly laparotomy or laparoscopy. Since giant cysts often adhere to surrounding tissues, they are difficult to approach laparoscopically.^5^ Additionally, it has been reported that laparoscopic surgery is not indicated for suspected malignant cysts considering the possibility of dissemination by cyst puncture.^6^ However, some reports mention that it is safe; therefore, a consensus has not been reached.^7^, ^8^

Because preoperative images could not rule out malignancy, we decided that total resection was needed. Generally, the difficulty of laparoscopic surgery for adrenal tumors increases with tumor size due to poor visibility and maneuverability. Comparatively, robot‐assisted surgery has great merits because it provides a clearer close‐up view, finer operation, and countertraction with the third arm. However, in the absence of a sense of touch, robotic surgery requires constant forceps manipulation awareness based on visually obtained information to avoid rupturing the cyst. We believe these merits are significant for cases in which malignancy cannot be ruled out or for adrenal cysts and tumors >6 cm. Following the adrenal myelolipoma removal by robot‐assisted surgery,^9^ ours is the second case of an adrenal tumor >6 cm.

The classification developed by Foster^9^ is widely used for the pathological classification of adrenal cysts, including parasitic (7%), epithelial (9%), endothelial (45%), and pseudocysts (39%). Pseudocysts are the most common resected adrenal cysts; causative factors are bleeding, infarction, and cystic degeneration from lesions such as normal adrenal tissue and tumors.^10^ The solid component on MRI was considered a clot based on the cut surface of the cyst and pathological findings. This case was conceived as an infection developed in an old encapsulated hemorrhagic pseudocyst. A much larger sample size and control group are needed to assess the safety and accuracy of this robot‐assisted surgery technique.

## Conclusion

We report a case of a giant adrenal pseudocyst successfully removed by robot‐assisted surgery. The merits of robot‐assisted adrenalectomy are considered significant for adrenal cysts and tumors >6 cm, for which malignancy cannot be ruled out.

## Author contributions

**Takayuki Ohzeki:** Writing – original draft; writing – review and editing. **Hiroyuki Kita:** Supervision. **Remon Kunishige:** Supervision. **Taiji Hayashi:** Supervision. **Tsukasa Nishioka:** Supervision.

## Conflict of interest

The authors declare no conflict of interest.

## Approval of the research protocol by an Institutional Reviewer Board

No ethical approval was required for this case report.

## Informed consent

Verbal informed consent has been obtained and is documented in the medical record.

## Registry and the Registration No. of the study/trial

Not applicable.
